# Characteristics of decline in cognition and locomotion among the elderly in seven provinces of China

**DOI:** 10.1002/agm2.12091

**Published:** 2019-12-20

**Authors:** Xiaoshuang Liu, Senlin Luo, Ping Zeng, Huan Gong, Yan Zhang, Enyi Zhang, Yiwen Han, Limin Pan, Jing Pang, Tiemei Zhang

**Affiliations:** ^1^ Information System and Security & Countermeasures Experimental Center Beijing Institute of Technology Beijing China; ^2^ The MOH Key Laboratory of Geriatrics National Center of Gerontology Beijing Hospital Beijing China

**Keywords:** Chinese elderly, cognition, gait speed, locomotion, MMSE

## Abstract

**Background:**

Decline in cognition and in locomotion is associated with aging. However, the relationship between them and the current occurrence of them in Chinese elderly people was weak.

**Methods:**

To investigate the details of these two functions in Chinese elderly people and to try to find some early recognition and intervention clues, data of MMSE test and usual gait speed from 4487 elderly people from seven provinces in China were analysed.

**Results:**

The prevalence of mild cognitive impairment (MCI) and dementia in persons aged 60 and over was 17.83% and 4.08%, respectively. Among 11 items of MMSE, calculation, three‐word recall, drawing two pentagons, and temporal orientation were the most commonly impaired items in persons with MCI or dementia. The gait speed of old persons with dementia was significantly slower than that of persons with MCI or NCI. Meanwhile, old persons with gait speed >1.39 m/s fast gait speed also had high MMSE scores and no dementia was detected by MMSE.

**Conclusion:**

The prevalence of dementia observed in this population was similar to that reported 20 years ago. Loss of temporal orientation and drawing two pentagons may supply more information for early recognition of cognitive impairment. Maintaining locomotion in a proper way may help old persons to prevent cognitive function decline.

## 1 INTRODUCTION

Maintenance of cognition and locomotion are the main elements for quality of life in old people. The impairment of these two functions can lead to the restriction of daily life,^1^, ^2^ accidental injuries,^3^ and death.^4^ During the process of aging, early recognition and intervention are essential to prevent functional decline. It is still hard to distinguish which kind of persons need to be trained and have early intervention for the two functional declines above.

Mini‐Mental State Examination (MMSE) is the most common and quickest way of assessing the status of cognition.^5^, ^6^ The total score of all items is always used to distinguish the stage of cognitive function decline. In addition to the scores, the impairment of different MMSE subsets had been analysed in the progression of dementia. Cognitive impairment is associated with physical function decline. Gait speed is the most extensively investigated aspect and had been proved to be closely related to cognitive function.^7^ However, most studies have focused on a single functional decline in aging or on the relationship between slow walking speeds and dementia;^8^ there has been little integrated research to assess these two functions and describe the details of their occurrence.

The objective of this study was to investigate the current situation regarding cognitive status and physical function in elderly Chinese people and to provide an integrated assessment of how these two functions decline with aging. Individual MMSE score, including analysis of subsets impairment, and usual gait speed have been analysed in community‐dwelling old persons from seven provinces in China.

## 2 METHODS

### 2.1 Study population

This study is constructed from a national survey of the Comprehensive Assessment of Elderly Health (CAEH) (a programme of the Research Special Fund for Public Welfare Industry of Health) in China. The CAEH survey was conducted in four provinces (Shanxi, Hunan, Sichuan, and Heilongjiang) and three municipalities directly under the Central Government (Beijing, Shanghai, Chongqing) during 2011 and 2012.

A multi‐stage stratified cluster sampling survey was designed to explore the general health situation of Chinese elderly people aged 60 or over. The subjects who live in local communities in these provinces and who did not have severe cardiopulmonary disease or crucial organ failure were encouraged to participate in the research. Interviewers were doctors and nurses from the hospitals and were trained for two weeks before the survey. The subjects' informed consent was obtained to use their information in this study; and the study was approved by the Ethics Committee of Beijing Hospital.

### 2.2 Definition of cognitive status

We classified cognitive status into three categories: no cognitive impairment (NCI), mild cognitive impairment (MCI) and dementia. The objective cognitive function was assessed by the 30‐point Mini‐Mental State Examination (MMSE).^9^ Participants were defined as having NCI if the score of the MMSE was >27. Participants were defined as having dementia if the score was <18 for illiterate subjects, or <21 for subjects with primary school degrees, or <23 for subjects with middle school degrees, or <24 for subjects with college or higher degrees. The detection criteria of MCI were between NCI and dementia for subjects with different educational levels. In this study, MCI and dementia were sometimes grouped together as cognitive impairment (CI).

### 2.3 Examination of gait speed

Gait speed was measured by the six‐metre walk test. Participants walked from a standing start and continued walking past the six‐metre end line at their usual pace. The total time from beginning to end was measured with a stopwatch. Gait speed was calculated by dividing the distance in metres by the time in seconds (m/s). The speed of more than 1.39 m/s was used to define high gait speed in the elderly.^10^

### 2.4 Data analysis

All data analyses were carried out using RStudio (an open‐source development environment for R, a programming language for statistical computing and graphics) and SPSS. Chi‐square tests were conducted to examine differences in the prevalence of MCI and dementia between males and females and differences in non‐full marks rates of MMSE items. Multiple chi‐square tests were used to determine the statistical difference in non‐full marks rate of any two MMSE items among particular cognitive status groups, a stricter *P*‐value threshold was set under Bonferroni correction.

To observe the role of age and education, the values of 60‐69 group and the illiterate group were used as basic values. The number of other groups was normalized relative to the basic value. And the adjusted ratio was used to observe the trend of cognitive function and locomotion activity altering with age or education:

Full marks rate = the amount of full marks persons/total participants number;
Adjusted ratio (Figure 2A) = full marks rate of each item in each age group/full marks rate of each item in the 60–69 group;
Adjusted ratio (Figure 2B) = full marks rate of each item in each education group/full marks rate of each item in the illiterate group;
Adjusted ratio for MMSE scores (Figure 4) = average MMSE scores of each age group/average MMSE scores of 60–69 group.
Adjusted ratio for gait speed (Figure 4) = average gait speed of each age group/average gait speed of 60–69 group.

## 3 RESULTS

### 3.1 Demographic features of study participants

Out of 4750 subjects targeted for the MMSE screening, 4487 subjects completed the interview (94.46% response rate). Characteristics of participants are shown in Table 1.

**Table 1:** Characteristics of participants

Factor	Group	Age (Mean ± SD)	Gender (Male/Female)	Number	Proportion (%)
	Total	70.56 ± 7.00	2030/2457	4487	100.00
Gender	Male	71.23 ± 7.08	2030/0	2030	45.24
	Female	70.00 ± 6.89	0/2457	2457	54.76
Age	60‐	64.24 ± 2.80	879/1219	2098	46.76
	70‐	74.31 ± 2.74	881/991	1872	41.72
	80‐	82.61 ± 2.91	270/247	517	11.52
Education	Illiterate	75.42 ± 6.93	91/305	396	8.83
	Primary school	70.53 ± 6.88	335/594	929	20.70
	Middle school	69.00 ± 6.67	760/957	1717	38.27
	College or higher	71.09 ± 6.80	844/601	1445	32.20
Current dwelling	Large and medium cities	71.09 ± 6.91	1518/1796	3314	73.86
	County towns	69.04 ± 6.86	148/171	319	7.11
	Rural area	69.09 ± 7.13	362/482	844	18.81

### 3.2 Prevalence of MCI and dementia by age, gender and education

Of 4487 study participants, 800 (17.83%) participants were suspected of MCI based on their performance on MMSE and 183 (4.08%) met the diagnostic criteria for dementia. The prevalence of MCI, dementia and CI by age and gender are shown in Table 2.

**Table 2:** Prevalence of MCI, dementia and CI by age and gender

Age group	Male	Female	MCI					Dementia					CI		
			Diagnosis (n)		Prevalence (%)			Diagnosis (n)		Prevalence (%)			Prevalence (%)		
			Male	Female	Male	Female	Total	Male	Female	Male	Female	Total	Male	Female	Total
60‐	444	695	45	98	10.14	14.10	12.55	6	20	1.35	2.88	2.28	11.49	16.98	14.84
65‐	435	524	27	74	6.21	14.12	10.53	8	8	1.84	1.53	1.67	8.05	15.65	12.20
70‐	429	546	60	125	13.99	22.89	18.97	13	18	3.03	3.30	3.18	17.02	26.19	22.15
75‐	452	445	82	124	18.14	27.87	22.97	18	35	3.98	7.87	5.91	22.12	35.74	28.87
80‐	270	247	73	92	27.04	37.25	31.91	15	42	5.56	17.00	11.03	32.60	54.25	42.94
Total	2030	2457	287	513	14.14	20.88	17.83	60	123	2.96	5.00	4.08	17.10	25.88	21.91

Abbreviations: CI, cognitive impairment; MCI, mild cognitive impairment.

Cognitive function was markedly affected by age and education. The prevalence of MCI and dementia in the 80+ years group was much higher than in the 60‐64 years group (MCI: 31.91% vs 12.55%; dementia: 11.03% vs 2.28%; Table 2). Among all the age groups, the prevalence of dementia was lowest in the college or higher group and highest in the illiterate group (0.16% and 15.28% for the 60‐69 years group; 1.2% and 15.96% for 70‐79 years group; 3.11% and 27.93% for 80+ years group; Table 3).

**Table 3:** Prevalence of MCI and dementia by education, age and gender

Education	Age group	Male	Female	MCI						Dementia					
				Diagnosis (n)		Prevalence (%)			*P* *	Diagnosis (n)		Prevalence (%)			*P* *
				Male	Female	Male	Female	Total		Male	Female	Male	Female	Total	
Illiterate	60‐	11	61	5	37	45.45	60.66	58.33	.825	1	10	9.09	16.39	15.28	.934
	70‐	49	164	22	96	44.90	58.54	55.40	.432	7	27	14.29	16.46	15.96	.927
	80‐	31	80	19	43	61.29	53.75	55.86	.839	4	27	12.90	33.75	27.93	.139
	Total	91	305	46	176	50.55	57.70	56.06	.584	12	64	13.19	20.98	19.19	.216
Primary school	60‐	114	309	24	84	21.05	27.18	25.53	.380	3	10	2.63	3.24	3.07	1.000
	70‐	165	237	56	92	33.94	38.82	36.82	.560	9	14	5.45	5.91	5.72	1.000
	80‐	56	48	28	24	50.00	50.00	50.00	1.000	2	6	3.57	12.50	7.69	.228
	Total	335	594	108	200	32.24	33.67	33.15	.805	14	30	4.18	5.05	4.74	.680
Middle school	60‐	418	568	38	47	9.09	8.27	8.62	.765	9	8	2.15	1.41	1.72	.532
	70‐	261	329	41	46	15.71	13.98	14.75	.696	10	9	3.83	2.74	3.22	.624
	80‐	81	60	12	12	14.81	20.00	17.02	.647	8	5	9.88	8.33	9.22	1.000
	Total	760	957	91	105	11.97	10.97	11.42	.616	27	22	3.55	2.30	2.85	.174
College or higher	60‐	336	281	5	4	1.49	1.42	1.46	1.000	1	0	0.30	0.00	0.16	1.000
	70‐	406	261	23	15	5.67	5.75	5.70	1.000	5	3	1.23	1.15	1.20	1.000
	80‐	102	59	14	13	13.73	22.03	16.77	.356	1	4	0.98	6.78	3.11	.134
	Total	844	601	42	32	4.98	5.32	5.12	.873	7	7	0.83	1.16	0.97	.717
Total		2030	2457	287	513	14.14	20.88	17.83	<.001	60	123	2.96	5.01	4.08	.001

Abbreviation: MCI, mild cognitive impairment.

* *P*‐values based on chi‐square test of independence for sex.

The prevalence of MCI or dementia was higher in women for almost every age group (Table 2). However, after adjusting for education and age, no significant gender difference was observed, as shown in Table 3.

### 3.3 Characteristics of cognitive function decline

**Figure 1:**
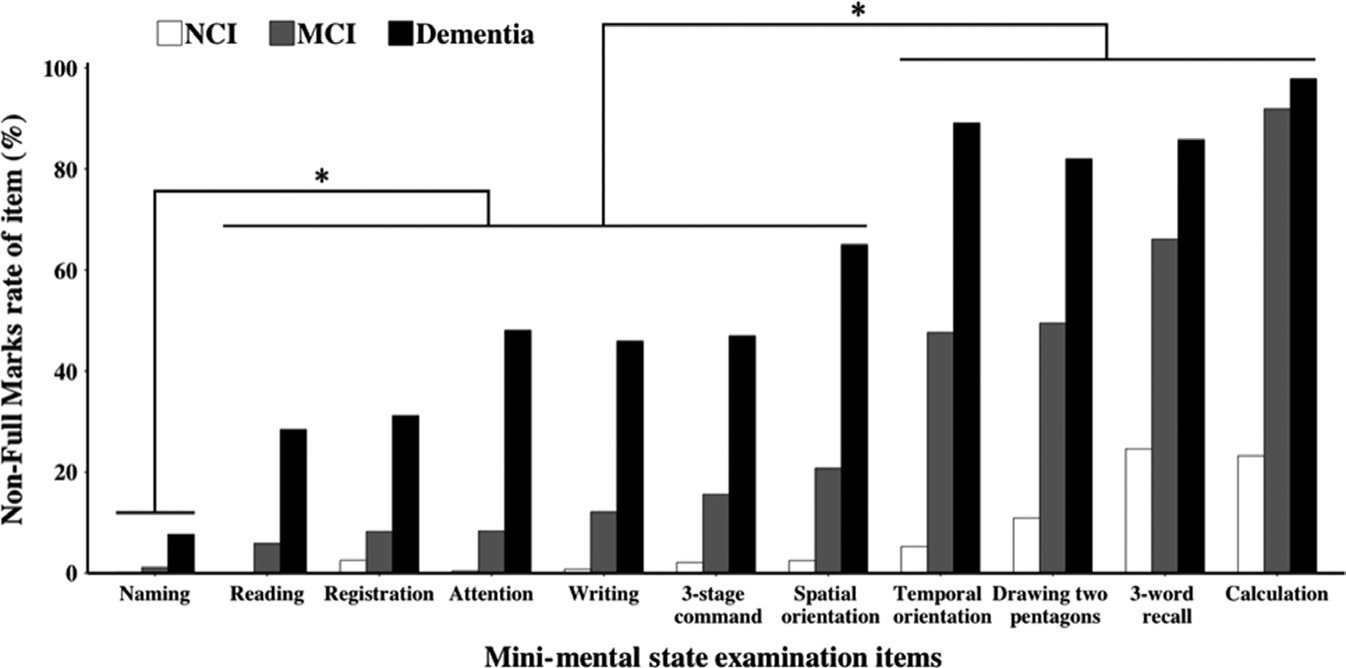
Non‐full marks rate of elderly people in specific items among three groups with different cognitive status. N = 4487. Non‐full marks rate is the proportion of elderly people who did not get full marks in specific item among particular cognitive status group. Multiple sets of Pearson chi‐square were used to determine the statistical difference of non‐full marks rate of any two MMSE items among a particular cognitive status group ($\alpha =0.05/C_{11}^{2}\approx 0.0009$) and the non‐full marks rate of items in different subgroups divided by the results had significant differences in each cognitive status group

Among 4487 study subjects, 2732 (62.16%) participants did not get full marks in the MMSE test. The proportion of persons who did not get full marks for each item is shown in Figure 1. According to the results of the chi‐square test, 11 items were divided into three groups. Calculation, three‐word recall, drawing two pentagons and temporal orientation formed the first group which had high non‐full marks rate in both MCI and dementia. The second group contained spatial orientation, three‐stage command, writing, attention, registration and reading. The loss of score in these items was easily detected in dementia compared to that in MCI. The third group is naming, which was hardly influenced in all participants.

### 3.4 Role of age and education for each MMSE item

**Figure 2:**
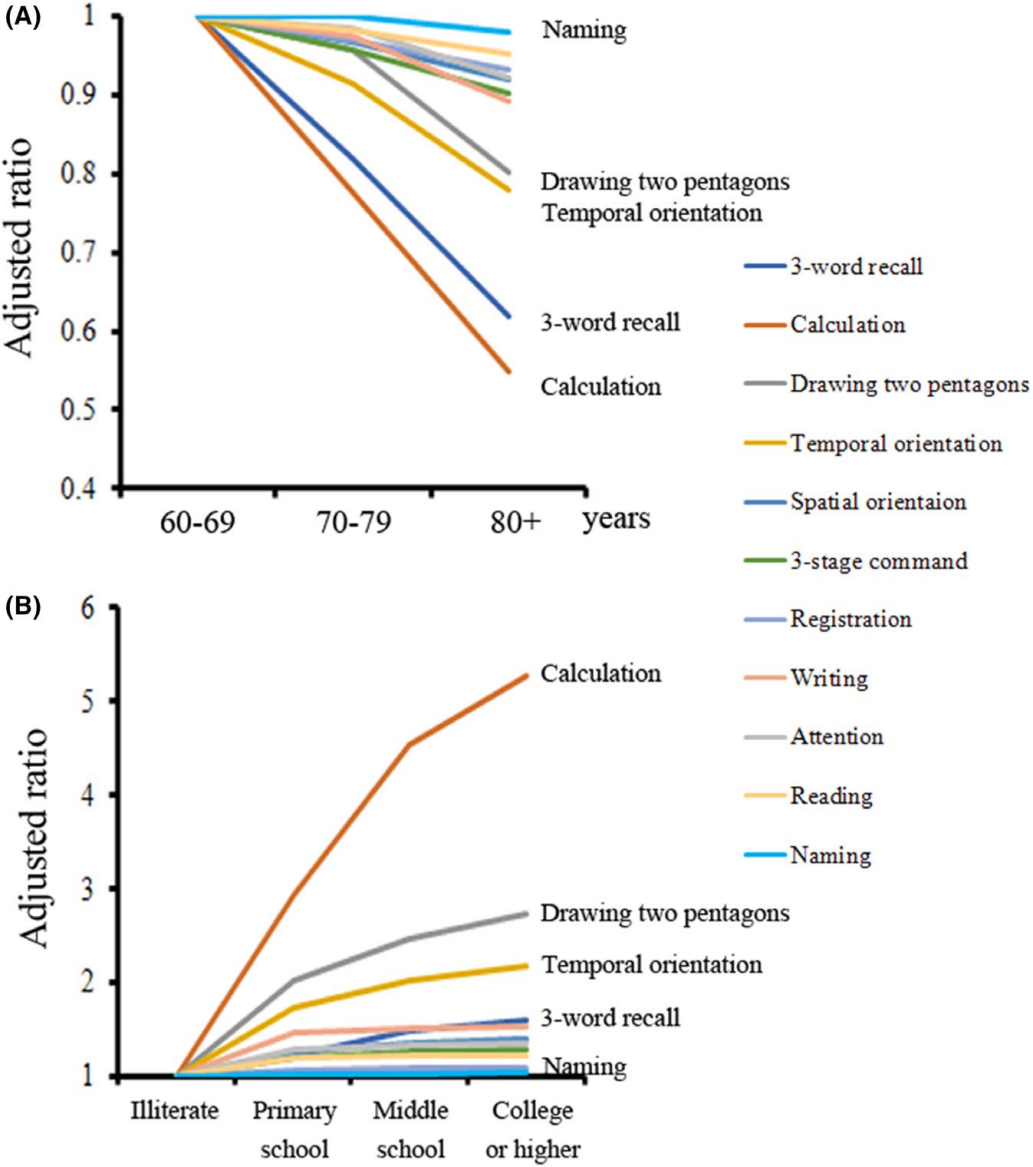
The role of age and education on MMSE items. A, the full marks rate of items in each age group was calculated relative to that in 60‐69 age group. B, the full marks rate of items in each education group was calculated relative to that in the illiterate group

Age and education were two important factors involved in the modulation of cognitive function. The full marks rate for each item was calculated in 60‐69, 70‐79 and 80+ age groups, and had a significant decrease with age. The full marks rate of each age group was normalized relative to the amount of 60‐69 group in each item. After adjustment, the full marks rate of calculation and three‐word recall declined rapidly with age compared to that of other items (Figure 2A). The full marks rate of each item was much higher in the college group than that in the illiterate group, as shown in Figure 2B. And the calculation item was modulated by the education most markedly among all the items; however, there was little role for education in the three‐word recall item which also decreased significantly with age (Figure 2B).

### 3.5 The relationship between cognitive status and gait speed

**Figure 3:**
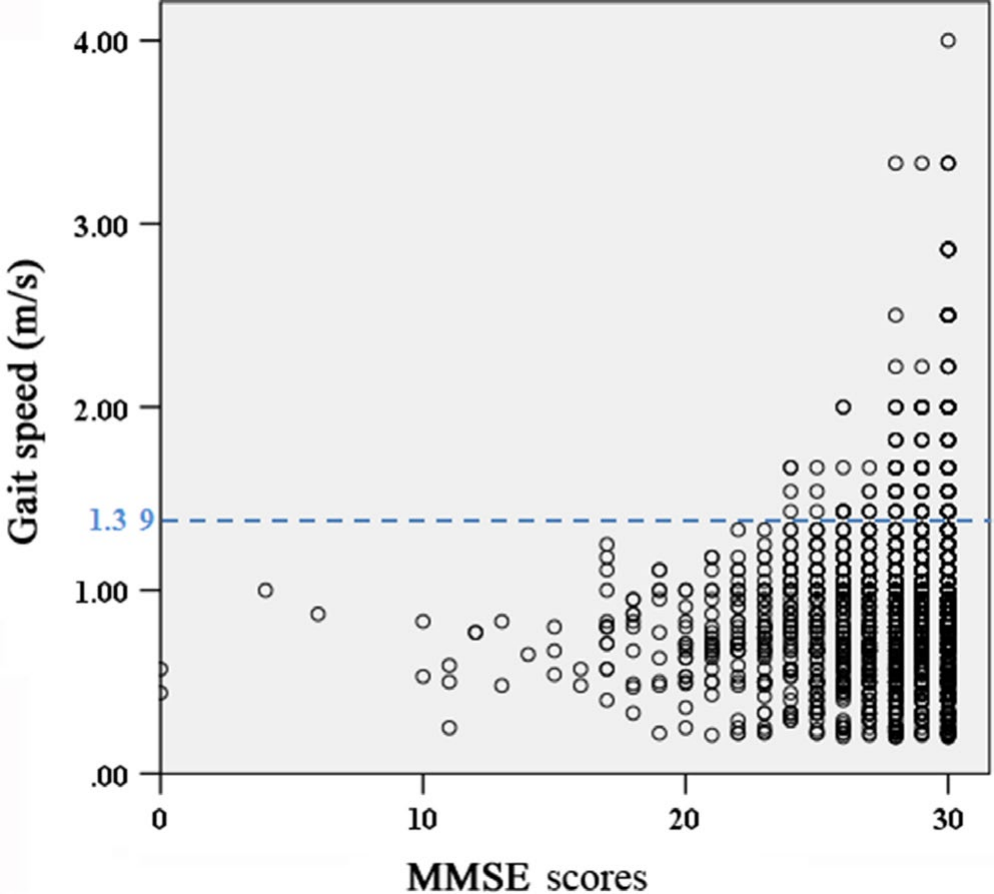
The relationship between MMSE scores and gait speed. The six‐metre gait speed was measured in 2819 participants. All participants were divided into two groups according to gait speed (GS > 1.39 m/s or not); and no dementia was detected in fast gait speed persons. The education levels of GS > 1.39 m/s participates were shown in the figure

Out of the 4487 study participants, 2819 took the walking speed test over a distance of six metres. The gait speed of persons in the dementia group (0.72 ± 0.23 m/s) was significantly lower than that of MCI (0.82 ± 0.32 m/s, *P* < .05) and NCI group (0.98 ± 0.4 m/s, *P* < .05, Table 4). Meanwhile, there was significantly different gait speed between different age groups (60‐69 years group: 0.91 m/s; 70‐79 years group: 0.82 m/s; 80+ years group: 0.74 m/s) and different educational groups (illiterate: 0.75 m/s; primary school: 0.86 m/s; middle school: 0.92 m/s; college or higher: 1.03 m/s; Table 4). Gait speed with 1.39 m/s was considered as the cut‐off value for fast gait speed in the elderly.^10^ There were about 8% of persons who had fast gait speed (>1.39 m/s) out of a total of 2819 participants and none of them had dementia (Figure 3). In addition, the prevalence of MCI in fast gait speed persons was only 6.6%, which was much lower than that in the total populations (17.83%, Table 5). Age and education level were considered in the fast gait speed group. The prevalence of MCI in participants with GS > 1.39 m/s was significantly less compared to that in the total participants in any education group (Table 5).

**Table 4:** Gait speed of dementia, MCI and NCI persons

	Group	Gait speed (m/s)			
		NCI	MCI	Dementia	Total
Age (y)	60‐69	1.02 ± 0.4	0.87 ± 0.3	0.84 ± 0.25*	0.91 ± 0.39
	70‐79	0.94 ± 0.41	0.81 ± 0.35	0.7 ± 0.21*	0.82 ± 0.4
	80+	0.83 ± 0.21	0.76 ± 0.26	0.64 ± 0.27*	0.74 ± 0.27
Education	Illiterate	0.82 ± 0.27	0.73 ± 0.26	0.71 ± 0.2	0.75 ± 0.26
	Primary school	0.88 ± 0.35	0.82 ± 0.31	0.74 ± 0.29	0.86 ± 0.34
	Middle school	0.93 ± 0.37	0.85 ± 0.33	0.71 ± 0.22*	0.92 ± 0.35
	College or higher	1.04 ± 0.41	0.84 ± 0.35	0.75 ± 0.27*	1.03 ± 0.41
Total		0.97 ± 0.4	0.82 ± 0.32	0.72 ± 0.23*	0.95 ± 0.39

Abbreviations: NCI, no cognitive impairment; MCI, mild cognitive impairment.

* *P* < .05 vs NCI.

**Table 5:** The prevalence of MCI in participants with gait speed > 1.39 m/s

Education	GS > 1.39 m/s participants				All participants	
	Total n	Age y	MCI %	Dementia %	MCI %	Dementia %
Illiterate	0	**/**	**/**	**/**	56.06	19.19
Primary school	18	69.1 ± 5.9	16.67	0	33.15	4.74
Middle school	79	67.1 ± 5.0	8.86	0	11.42	2.85
College or higher	129	68.3 ± 5.8	3.88	0	5.12	0.97
Total	226	67.9 ± 5.6	6.64	0	17.83	4.08

Abbreviations: GS, gait speed; MCI, mild cognitive impairment.

**Figure 4:**
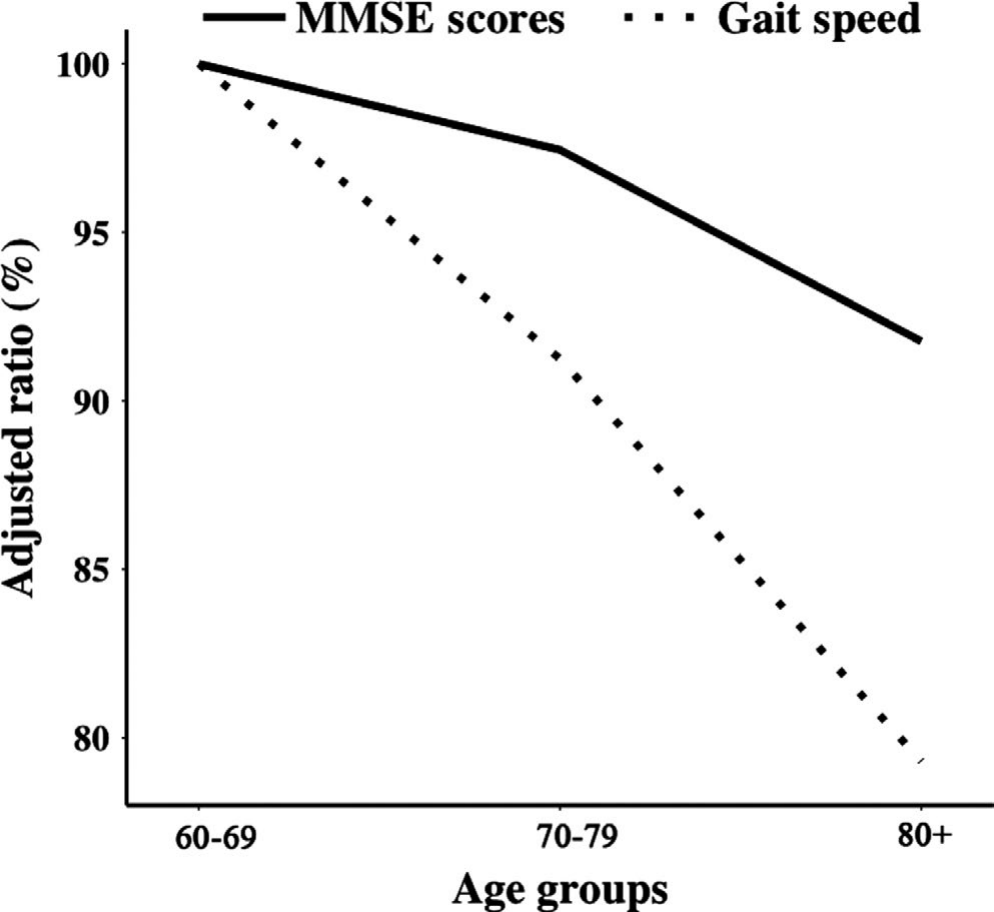
The decline rate of MMSE scores and gait speed with age. Both the MMSE scores and gait speed of each age group were calculated relative to those of 60‐69 age group

To compare the trend of cognition and locomotion changing with age, we calculated the relative values of MMSE scores and gait speed in all age groups to the values in 60‐69 group. After adjustment, it seemed that the decline rate of gait speed was more obvious than that of MMSE scores with age. And the decreasing speed of both of them accelerated with aging (Figure 4).

## 4 DISCUSSION

The MMSE is the most widely practised screening test for identifying cognitive impairment in large community‐based populations. It consists of 11 separate items, which are allocated to different subtests and which represent cognitive domains. In this study, we used MMSE and gait speed to evaluate the cognitive and locomotive function of Chinese elderly adults from seven provinces. The results of this study indicated that: (a) the prevalence of MCI and dementia in persons 60 years and over is 17.83% and 4.08%, respectively; (b) cognitive function decline has a strong relationship to age and education; (c) calculation, three‐word recall, drawing two pentagons, and temporal orientation were four highly impaired items in the MCI and dementia groups; and (d) persons with fast gait speed also had high MMSE scores, and no dementia was detected by MMSE in old persons with fast gait speed.

In this study, the gender difference in the prevalence of MCI and dementia was eliminated after adjusting by education. This suggested that age and low education were independent risk factors for dementia, which was consistent with previous studies.^11^, ^12^ The prevalence of dementia in persons of 65 and older was 4.7%, similar to the values (4.6%) published earlier from Shanghai in 1990 and the prevalence of AD (4.8%) in four regions of China in 1997.^12^, ^13^ Recently, Jia et al.^14^ had reported that in 2009 the prevalence of dementia was 5.14% in 30 urban and 45 rural communities. Compared with the obviously increase of prevalence in common NCD (hypertension, diabetes) in China, the prevalence of dementia did not change significantly during these 20 years. This suggested that older age is the strongest risk factor for dementia, but not lifestyle. As the Chinese population is aging rapidly, the number of people with dementia is increasing year by year. It is crucial for us to explore effective ways of early recognition of dementia and intervention.

To identify the characteristics of early cognitive function decline, we analysed the distribution of the impairment in MMSE subsets among NCI, MCI and dementia. Previous follow‐up investigations had revealed that in MCI, orientation for time was the specific MMSE domain associated with cognitive decline after controlling for age, sex, and education.^15^, ^16^ In 2018, WHO indicated that time orientation and three‐word recall were two important factors for cognition assessment. Here, in our data, a loss score in temporal orientation and three‐word recall also got high frequency in persons with impairment of cognitive function.

Walking speed is a summary indicator of frailty, which can be influenced by changes of central nervous, cardiorespiratory, musculoskeletal systems and so on.^17^ Previous studies showed a significant association between poorer cognitive function and slower gait speed.^8^ Consistent with these, the gait speed of dementia was significantly slower compared to that of MCI and NCI participants in our work. A walking speed <0.8 m/s was used to define mobility impairment.^18^ Here, we used 1.39 m/s as a fast gait cut‐off point to divide the participants into two groups; and there was no dementia detected in elderly persons with this gait speed. This may suggest that the cut‐off value for fast gait speed could be an indicator applied for cognitive function evaluation. As an objective measurement, walking speed is amenable to assessment and intervention.

Cognition and locomotion were put together to observe their trend of declining with age. The data told us that intervention with physical activity should be conducted earlier in the elderly and retaining locomotive activity may help people prevent cognitive function decline.

## CONFLICTS OF INTEREST

Nothing to disclose.

## AUTHOR CONTRIBUTIONS

*Writing of paper*: all authors. *Design, literature review and coordination*: Zhang TM and Pang *Data cleansing, statistical analysis, literature review*: Liu, Luo and Pan. *Data *collection: Zeng, Gong, Zhang Y, Zhang EY and Han.
